# COVID-19 in northeast Brazil: first year of the pandemic and uncertainties to come

**DOI:** 10.11606/s1518-8787.2021055003728

**Published:** 2021-05-21

**Authors:** Ligia Regina Franco Sansigolo Kerr, Carl Kendall, Rosa Lívia Freitas de Almeida, Maria Yury Ichihara, Estela Maria L Aquino, Antônio Augusto Moura da Silva, Ricardo Arraes de Alencar Ximenes, Maria de Fatima Pessoa Militão de Albuquerque, Naomar Almeida-Filho, Rafael Felipe Souza, Sinval Pinto Brandão, Wayner Vieira de Souza, Maurício Lima Barreto

**Affiliations:** I Universidade Federal do Ceará Faculdade de Medicina Departamento de Saúde Comunitária FortalezaCE Brasil Universidade Federal do Ceará. Faculdade de Medicina. Departamento de Saúde Comunitária. Programa de Pós-Graduação em Saúde Coletiva. Fortaleza, CE, Brasil; II Tulane University School of Public Health and Tropical Medicine New Orleans USA Tulane University School of Public Health and Tropical Medicine. New Orleans, LA 70112, USA; III Universidade de Fortaleza Programa de pós-graduação em Saúde Coletiva FortalezaCE Brasil Universidade de Fortaleza. Programa de pós-graduação em Saúde Coletiva. Fortaleza, CE, Brasil; IV Fundação Oswaldo Cruz Centro de Integração de Dados e Conhecimentos para Saúde Instituto Gonçalo Moniz SalvadorBA Brasil Fundação Oswaldo Cruz. Centro de Integração de Dados e Conhecimentos para Saúde Instituto Gonçalo Moniz. Salvador, BA, Brasil; V Universidade Federal da Bahia Instituto de Saúde Coletiva SalvadorBA Brasil Universidade Federal da Bahia. Instituto de Saúde Coletiva. Salvador, BA, Brasil; VI Universidade Federal do Maranhão Centro de Ciências da Saúde Departamento de Saúde Pública São LuísMA Brasil Universidade Federal do Maranhão. Centro de Ciências da Saúde. Departamento de Saúde Pública. São Luís, MA, Brasil; VII Universidade de Pernambuco Faculdade de Ciências Médicas RecifePE Brasil Universidade de Pernambuco. Faculdade de Ciências Médicas. Recife, PE, Brasil; VIII Universidade Federal de Pernambuco Centro de Ciências da Saúde RecifePE Brasil Universidade Federal de Pernambuco. Centro de Ciências da Saúde. Recife, PE, Brasil; IX Fundação Oswaldo Cruz Instituto Aggeu Magalhães RecifePE Brasil Fundação Oswaldo Cruz. Instituto Aggeu Magalhães. Recife, PE, Brasil

**Keywords:** Coronavirus Infections, epidemiology, Communicable Diseases, Emerging, prevention & control, Pandemics, Epidemiology, Descriptive

## Abstract

**OBJECTIVE:**

To analyze the epidemic of COVID-19 in northeastern Brazil, one of the regions most affected by the virus.

**METHODS:**

The official data for COVID-19, from March 2020 to March 2021 in the states of the Northeast Region (NE), were used. The analysis of capital cities and states for accumulated weekly cases and confirmed deaths was made using the JoinPoint Trend Analysis application.

**RESULTS:**

In one year, the Northeast region reported 22.9% of the cases and 21.5% of the deaths in the country due to COVID-19. At the beginning of the pandemic, all states showed a growing number of cases, first in the capitals and then in the interior. Following this wave, decreases are observed in all states and their capitals, but with many still reporting a large number of cases. In the middle of the 2nd semester of 2020 the number of cases begins to increase again simultaneously in states and their capitals—some at explosive speed—especially in late 2020 and early 2021. A similar pattern is observed in deaths, which exceed or approach the peak seen in the first wave. In the first wave, all capitals and northeastern states adopted intense isolation measures. Fortaleza, Recife and Teresina reached the highest isolation index of all capitals, close to 0.60. This index decreases, with a slight growth trend until the end of December. With the exception of Fortaleza and Salvador, the other capitals fell to less than 0.40.

**CONCLUSION:**

The Brazilian NE and the country are in increasingly complicated health, social and economic situations. It is necessary to speed up vaccinations and maintain non-pharmacological measures: face masks, social distancing measures and hygiene care, in addition to policies to protect workers who have lost their incomes and to subsidize small business owners.

## INTRODUCTION

The COVID-19 pandemic in Brazil is expanding and is sending worrying signals to the world about what will happen in the coming weeks and months in Brazil. Although Brazil represents only 2.7% of the world population, the country is responsible for 13.3% of all COVID-19 cases and 26.9% of deaths as of March 29, 2021^1,2^. The largest unitary health system in a developing country is failing to cope with the tragedy that has befallen the country. In January 2021, some states recorded a greater number of cases in a single day than the worst day in the first wave of COVID-19 in 2020^3^. Viruses, especially RNA viruses, produce mutations, however rapid mutation is not associated with the common coronaviruses that cause colds in humans. Recently, more specifically in late 2020 and early 2021, new variants of SARS-COV-2 were detected in the United Kingdom^4^, South Africa^5^and Brazil^6^.

The Northern Region of Brazil was the most affected by the first and second waves of the pandemic. In a very short time, in the capital of Amazonas, Manaus, the strain called P1^6^, with greater transmissibility, spread quickly, dominating the scenario—from 31% of the strains of the examined samples, in December 2020, to 91% of the strains present in examined samples , in January 2021^7^.

The state of Amazonas anticipated what occurred in other regions of the country, particularly the Northeast, which along with the North Region, is among the poorest in the country. In Manaus, when patients died for lack of hospital oxygen after a history of unanswered requests to state and federal authorities, the country and the world were appalling.

Mask use and social distancing, including closing venues such as schools and universities, prohibiting mass events, restricting travel and public transport, encouraging home isolation, or even the complete prohibition of circulation on the streets except for the purchase of food, medications and health care, are still essential^8^. Still, vaccines are, to date, the most promising strategy since the beginning of the epidemic. Data from some developed countries that managed to achieve high rates of vaccine coverage using a variety of strategies, such as Israel, obtained encouraging initial results. They suggest that, even with coverage that does not reach 100% of the population, vaccines produce marked reductions in severe cases and infections. In Israel, the infection rate among those adequately vaccinated (that is, one week after taking two doses of the vaccine against the disease) was 0.04%. Of the vaccinated people who were infected, only 16 had to be treated in hospital—0.002% of all cases^9^.

The objective of this study was to update the situation of the COVID-19 epidemic in the Northeast Region of Brazil and to discuss the epidemic in the face of political and economic contexts, viral evolution, measures of social distancing, and vaccine availability.

## METHODS

The study reviewed data for the nine states in northeastern Brazil, one of the poorest regions in the country. The data for the municipalities reference the period from the beginning of the epidemic, March 2020, to the second half of March 2021. The confirmed Covid-19 cases and deaths are reported by the Ministry of Health and State Secretariats, available in an open data repository^10^.

Cases and deaths were analyzed for states and their capital municipal areas separately by epidemiological week. Analysis of the series of weekly cases and confirmed COVID-19 deaths were facilitated using JoinPoint Trend Analysis^11^. To describe linear trends over time, the estimated annual percentage change ( Annual Percentage Change - APC) was calculated for each trend by adjusting a regression line to the natural logarithm of the number of cases, using the epidemiological week as the unit of time. A negative APC means a decrease in the number of cases, while a positive result denotes an increase. The results presented a statistically significant change in the trend if the results of the estimated regression coefficients for the difference in the slopes had a P value lower than 0.05^11^.

To analyze the daily percentage of the population of the municipalities and states that remain at home, the social isolation index, constructed by InLoco^®^, was used based on daily geolocation data captured on mobile devices as of February 1, 2020^12^.

## RESULTS

For the 64 reviewed weeks of the pandemic, the Northeast recorded 2,922,126 cases of COVID-19 and 69,009 confirmed deaths (03/31/2021), representing 22.9% of the cases and 21.5% of the deaths in Brazil, presenting great variability across time and region.

**Figure 1:**
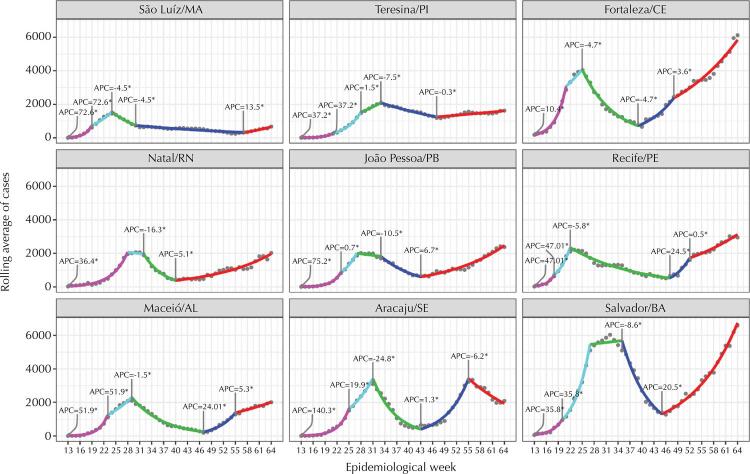
Confirmed cases of COVID-19 in the capitals of the nine states of the Northeast by period and statistically significant change over time, by epidemiological week, 2020.

**Figure 2:**
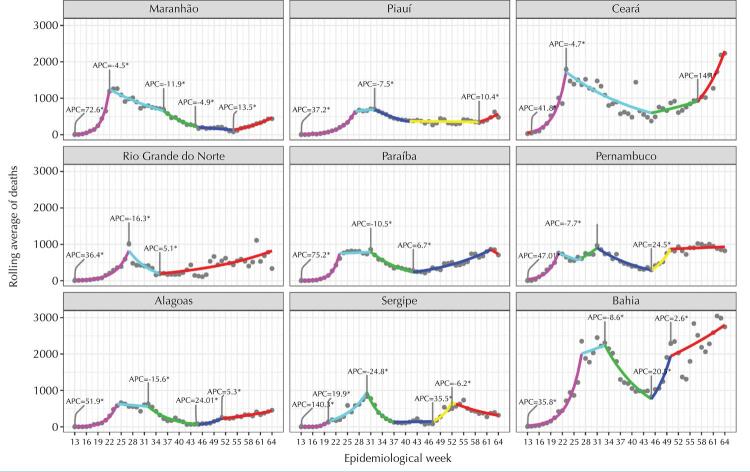
Confirmed cases of COVID-19 in the nine states of the Northeast by epidemiological week 2020 and statistically significant change over time.

**Figure 3:**
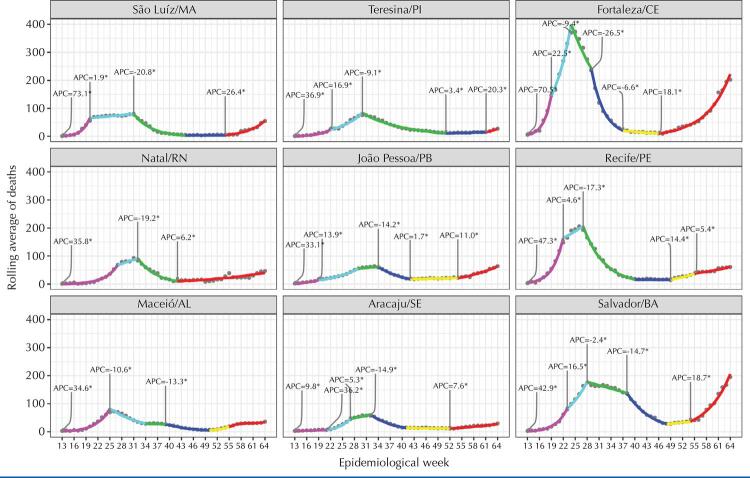
Deaths due to COVID-19 in the capitals of the nine states in the Northeast and statistically significant change over time, by epidemiological week, 2020.

**Figure 4:**
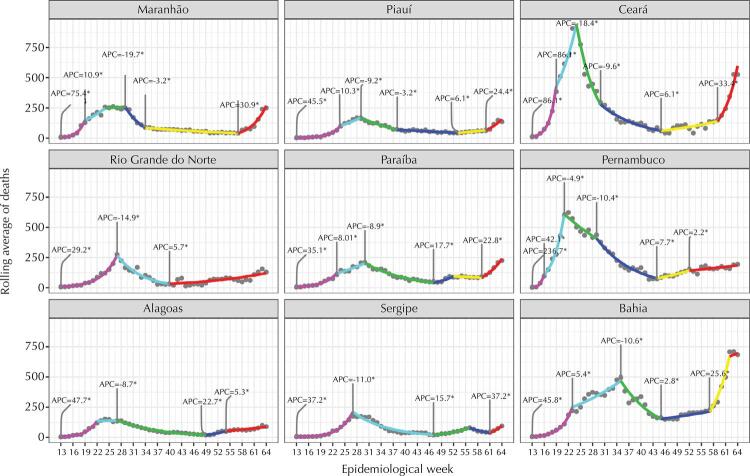
Deaths due to COVID-19 in the nine states in the northeast region by statistically significant change over time, by epidemiological week, 2020.

The trends in the number of confirmed and notified cases in the nine states of the region and their respective capitals (Figures 1 and 2), despite important variations, share an initial peak that occurred around the 17th week of the pandemic and a 2nd ongoing wave growing to new peaks that in most states and capitals already is, or will be, greater than the first. At the beginning of the pandemic, we observed that the states experienced a growth in the number of cases for about 6 weeks: first in the capitals, then, in the interior of each state. After, decreases are observed in all states and their capitals, but many states remained at a high level before the new wave of infection began. In the middle of the second half of 2020, the number of cases begins to increase in all states and capitals, some with explosive speed at the end of 2020 and the beginning of 2021, such as Ceará, Pernambuco and Bahia. Regarding deaths (Figures 3-4), the number has exceeded or is approaching the ceiling observed in the first wave, similar to the pattern of cases.

**Figure 5:**
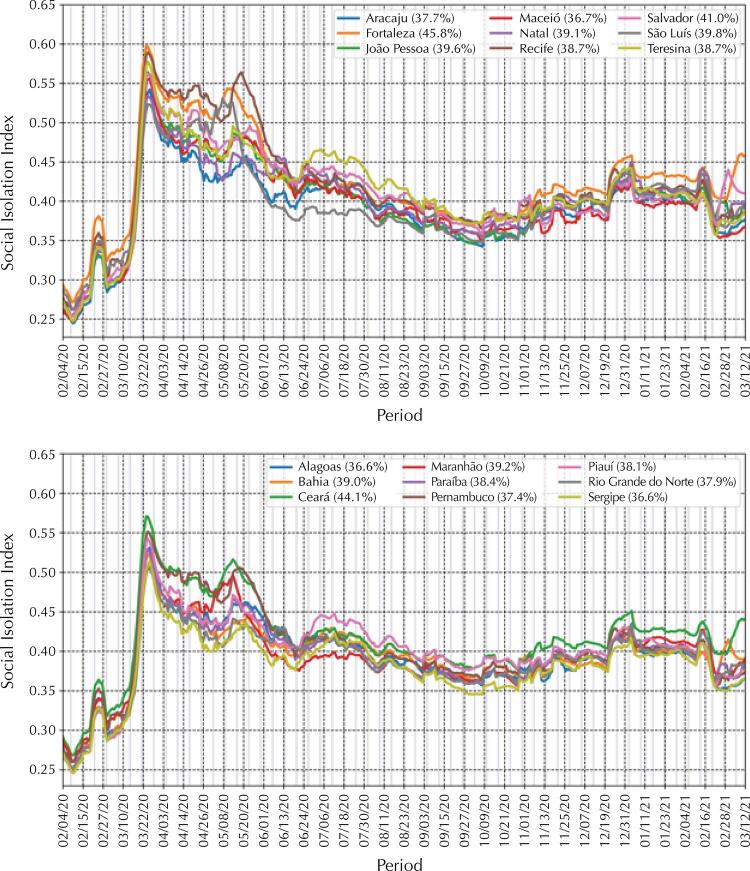
Social distancing measures by state and capital in Northeast Brazil.

In the first wave, all capitals and states in the northeast adopted comprehensive isolation measures, using the English term *lockdown*. Lockdown was decreed almost simultaneously in the nine states. The cities of Fortaleza, Recife and Teresina reached the highest isolation index of all capitals—close to 0.60. This was the only time during the pandemic that capitals and states managed to get close to these values. This isolation index then started to decrease, with some variations, reaching its minimum (0.35) around October 9, 2020. From then on, the isolation index has a slight growth trend reaching its peak around December 31, decreasing with a small peak of isolation in mid-February, and then decreasing again—with the exception of Fortaleza and Salvador, which remain with the isolation index above 0.40. All other capitals, since mid-February, have fallen below 0.40.

## DISCUSSION

At the beginning of 2021, several northeastern states started to report a sustained increase in the number of cases and deaths from COVID-19, some of which werealready reaching levels very close to or even higher than the first wave. In response to the first wave, the Governors and health authorities, in coordination, implemented comprehensive non-pharmacological measures and strengthened the health system, managing to mitigate the effects of the pandemic in an impoverished region before vaccines were available^13^. However, in the second wave, states and municipalities, although unified in their efforts to have the population vaccinated as soon as possible, acted with different strategies in other areas, the majority kept the economy open for a long time, even in the face of evidence of a worsening epidemic.

The opening of the economy in the second half of 2020, without the full range of non-pharmaceutical measures, is most likely the explanation of the current situation, which quickly became dramatic, especially for the most vulnerable populations in these states^14^. In November (epidemiological week 46), the elections—postponed by COVID-19^15^—occurred in the 5,570 Brazilian municipalities. The pre-election period and the election itself, with mandatory voting, promoted mass meetings, long lines and a greater possibility of spreading the virus^16^.

In addition to the economic opening, the elections, and end of the year partiesanother aggravating factor emerged: a new viral variant of Sars-Cov-2, called P1. The P1 strain was first detected in four travelers returning to Japan from the state of Amazonas on January 2, 2021^17^. It is estimated that it emerged in the capital of Amazonas in mid-November 2020, about a month before the number of hospitalizations for severe acute respiratory syndrome in the city exploded. The new strain accumulated 17 mutations, until it managed to “escape” the immunity of those who previously had the disease^18,19^. This would be the probable explanation of the large outbreak that occurred in Manaus earlier this year. It should be noted that Manaus had already suffered a very violent first wave and a large part of its population had been infected by the virus^20^. P1 is able to produce reinfections^6^, it is about 2.5^21^to 10 times more transmissible than the original strains^22^, and it can have a higher mortality rate than predecessor strains, much like the United Kingdom variant, whose mortality is 1.64 (95% CI: 1.34 - 2.04) times higher than the previous strain^23^.

The P1 variant, like others already described, increases the transmission velocity (Rt), causing the epidemic to have a faster rate of spread, reaching more people in a shorter period. It also replaces the previous strain in a short time—around 7 weeks. In Manaus, where an estimated attack rate of 70% in the first wave^24^did not prevent a new and devastating wave, led to the chaos reported internationally, in the already fragile local health system. No action recommended by experts to contain P1 was taken. On the contrary, since necessary assistance could not be provided to patients in Amazonas, they were transferred to several other states in the country without special safety precautions, which may have contributed to the dissemination of the new variant to various parts of the country.

The negationist stance of the Brazilian Federal Government contributed to the chaos in Manaus and to the rising number of cases inboth the Northeast and in more developed regions of the South^25,26^. A review of the policies produced by the current federal government related to the COVID-19 pandemic reveals an institutional strategy that, instead of seeking to control it, favors the spread of the virus in the country^27^. In fact, with the transmission highway paved by the lack of support for social distancing and mask use and the increase in transmission due to P1, the variant quickly spread to different areas of the country. A study released in early March 2021, conducted in eight Brazilian states, including three in the Northeast, found that 71.1% of the samples from Ceará, 50.8% from Pernambuco and 42.6% from Alagoas were the P1 variant^28^.

As preventive measures such as the use of masks, social distancing and hygiene protocols that began in the middle of the second half of 2020 are declining and new variants are spreading across Brazil, the extent of the tragedy is becoming increasingly evident, with the collapse or imminent collapse of the health system in several cities and in the Northeast Region. Five of the 27 capitals in the country have ICU occupancy rates equal to or greater than 80%, 15 of them already exceed 90%^29^and the situation only worsens. The overload and collapse of the health system is imminent and the worsening and “rejuvenation” of the epidemic in Brazil are visible. The Brazilian Association of Intensive Care Medicine compared deaths in the period between September and November 2020 with the present moment of collapse (between February 1 and March 26, 2021), which showed an 193% increase in deaths among 18 to 45-year-olds, and ICU occupancy by this cohort also increased from 13.1% to 38.5% in public and private hospitals in the country^30^.

The situation is further aggravated by the very low rates of vaccination coverage. Although Brazil is among one of the only countries that would be able to vaccinate 10 million people in a single day, due to the high quality and local penetration of its National Immunization Program, it has managed to vaccinate only 8% of the population^31^. Once again, the federal government not only did not buy the necessary vaccines, but also refused to organize the vaccination response. It should be noted that the government still belittles the value of the available vaccines. The Brazilian Government could have purchased or received around 316 million doses in the first half of 2021, of which 200 million would have come from COVAX^32^, the Global Vaccine Alliance created by 165 countries, which would be enough to vaccinate 78% of the population, but until now, only the second priority population, those 60-74 year of age, have began receiving the vaccine^33^. Even the small advances in vaccination demonstrated a decrease in mortality and severe cases among vaccinated older adults population (>75) in São Paulo (51%), Pernambuco (26%) and Ceará (46%)^34-36^. In an attempt to respond to the situation, and in a coordinated action by the Governors of the Northeast Consortium, 39 million doses of the Sputnik V vaccine, developed in Russia ^37^, were purchased. This vaccine is in the approval process for emergency use by the National Sanitary Surveillance Agency (ANVISA).

The scenario in Brazil is not good and the situation in northeast Brazil is alarming. The measures are unlikely to have a major impact if they are only dealt with regionally. Measures must be national and much more effective to face the worst health crisis Brazil has ever faced, at its most critical moment. Presenting some of the worst indicators of COVID-19 in the world, the current situation is certainly associated with the lack of effective and coordinated responses from the federal government. Brazil, despite presenting one of the most serious and critical national case studies of the pandemic in the world, has responded only a year after the beginning of the pandemic by creating a National Coordination Committee to Fight the COVID-19 Pandemic^38^. Innumerable doubts are raised about the real role of this committee in controlling the epidemic in the country, since it is ultimately led by a negationist president.

The National Mayors’ Front reports that Brazilians “are abandoned by the federal government”^39^, at the same time that the number of cases in Brazil is growing alarmingly. More than 300 thousand lives have been lost, of which 75% could have been avoided^40^. A system created to evaluate international COVID-19 pandemic management examined the response state of countries 36 weeks after the 100th confirmed case of the virus; comparing Brazil with the 97 other countries, Brazil was in the 98th position with the lowest score—4.8 out of 100^41^. Other aggravating factors, such as the growth of food insecurity due to the pandemic—which increased by 15.5% in families with children up to 9 years of age in the state of Ceará^42^—will make the social and economic situation more and more complex. There is an urgent need to implement measures to protect the most vulnerable populations, who are a large part of the people living in poverty.

Speeding up the vaccination process is an essential measure to reduce mortality, severe cases of the disease and even cases of COVID-19 in general. Given the slow pace of vaccination, containing the pandemic at an advanced stage before vaccination will require renewed efforts to maintain existing non-pharmacological measures: the constant use of face masks, social distancing measures and hygiene protocols, in addition to policies to protect workers who have lost their incomes and to subsidize small business owners.
