# Supplementary material for: COVID-19 in northeast Brazil: first year of the pandemic and uncertainties to come
Source: Rev Saude Publica. 2021 May 21;55:35. doi: 10.11606/s1518-8787.2021055003728 (PMC8139846; doi:10.11606/s1518-8787.2021055003728)
Supplement: Translation

# Translation: Covid-19 no Nordeste do Brasil: primeiro ano de pandemia e incertezas que estão por vir

**Authors:** Ligia Regina Franco Sansigolo Kerr, Carl Kendall, Rosa Lívia Freitas de Almeida, Maria Yury Ichihara, Estela Maria L Aquino, Antônio Augusto Moura da Silva, Ricardo Arraes from Alencar Ximenes, Maria de Fatima Pessoa Militão de Albuquerque, Naomar Almeida-Filho, Rafael Felipe Souza, Sinval Pinto Brandão, Wayner Vieira de Souza, Maurício Lima Barreto

**Affiliations:** I Brasil Universidade Federal do Ceará. Faculdade de Medicina. Departamento de Saúde Comunitária. Programa de Pós-Graduação em Saúde Coletiva. Fortaleza, CE, Brasil; II USA Tulane University School of Public Health and Tropical Medicine. New Orleans, LA 70112, USA; III Brasil Universidade de Fortaleza. Programa de pós-graduação em Saúde Coletiva. Fortaleza, CE, Brasil; IV Brasil Fundação Oswaldo Cruz. Centro de Integração de Dados e Conhecimentos para Saúde Instituto Gonçalo Moniz. Salvador, BA, Brasil; V Brasil Universidade Federal da Bahia. Instituto de Saúde Coletiva. Salvador, BA, Brasil; VI Brasil Universidade Federal do Maranhão. Centro de Ciências da Saúde. Departamento de Saúde Pública. São Luís, MA, Brasil; VII Brasil Universidade de Pernambuco. Faculdade de Ciências Médicas. Recife, PE, Brasil; VIII Brasil Universidade Federal de Pernambuco. Centro de Ciências da Saúde. Recife, PE, Brasil; IX Brasil Fundação Oswaldo Cruz. Instituto Aggeu Magalhães. Recife, PE, Brasil

**Keywords:** Infecções por Coronavírus, epidemiologia, Doenças Transmissíveis Emergentes, prevenção & controle, Pandemias, Epidemiologia Descritiva

## Abstract

**OBJETIVO:**

Analisar a epidemia da covid-19 na região Nordeste do Brasil, uma das mais atingidas por essa virose.

**MÉTODOS:**

Os dados oficiais para covid-19 dos estados do Nordeste são referentes ao período de março de 2020 a março de 2021. A análise para capitais e estados da série do acumulado semanal de casos e de óbitos confirmados foi feita com emprego do aplicativo JoinPoint Trend Analysis.

**RESULTADOS:**

Em um ano, o Nordeste acumulou 22,9% dos casos e 21,5% dos óbitos do país pela covid-19. No início da pandemia, todos os estados apresentaram um crescimento sustentável de casos, primeiro nas capitais e depois interior. Em seguida, observam-se decréscimos em todos os estados e suas capitais, porém muitos permanecem com números elevados. Em meados do segundo semestre de 2020, o número de casos começa a crescer nos estados e suas capitais, alguns em velocidade explosiva, em especial no final de 2020 e início de 2021. Padrão similar é observado para os óbitos, os quais ultrapassaram ou aproximam-se do teto observado na primeira onda, na qual todas as capitais e estados do Nordeste adotaram intensas medidas de isolamento. Fortaleza, Recife e Teresina atingiram os maiores índices de isolamento de todas as capitais, perto de 0,60. Esse índice decresce, com tendência leve de crescimento até final de dezembro, voltando a decrescer. Com exceção de Fortaleza e Salvador, as demais capitais caíram para menos de 0,40.

**CONCLUSÃO:**

O Nordeste brasileiro e o país estão em uma situação sanitária, social e econômica cada vez mais complexa. É necessário acelerar o processo de vacinação e manter as medidas não farmacológicas – constante uso de máscaras faciais, medidas de distanciamento e cuidados de higiene –, além de políticas de proteção aos trabalhadores que perderam as suas rendas e subsídios aos pequenos empresários.

## INTRODUÇÃO

A pandemia da covid-19 no Brasil encontra-se em expansão e dá sinais preocupantes do que acontecerá nas próximas semanas e meses. Embora represente 2,7% da população mundial, o país é responsável por 13,3% dos casos de covid-19 e 26,9% dos óbitos neste momento (29Mar2021)^1,2^. O maior sistema público de saúde entre os países em desenvolvimento não está conseguindo dar conta da tragédia que se abateu sobre o país. No mês de janeiro de 2021, alguns estados já registravam um número de casos maior em um único dia que o pior momento da primeira onda da covid-19 em 2020^3^. Os vírus, em geral, sob elevadas taxas de disseminação, podem apresentar várias mutações, embora não sejam conhecidas rápidas mutações nos coronavírus mais comuns, causadores de resfriados que afetam os seres humanos. Recentemente, mais especificamente no final de 2020 e início de 2021, novas variantes do SARS-COV-2 vêm sendo detectadas em alguns países, como Reino Unido^4^, África do Sul^5^ e Brasil^6^.

A região Norte do país foi a mais afetada pela primeira e, também, pela segunda onda da epidemia. Em pouquíssimo tempo, na capital do Amazonas, Manaus, a cepa denominada P1^6^, com maior transmissibilidade, rapidamente se disseminou, passando de 31% das cepas das amostras examinadas em dezembro de 2020 para dominar o cenário, com 91% das cepas presentes nas amostras examinadas de suspeitos em janeiro de 2021^7^.

O que ocorreu no estado do Amazonas serviu de alerta do que poderia ocorrer brevemente em outros estados da federação, em especial da região Nordeste, que, junto com a Norte, está entre as mais pobres do país. Em Manaus, o quadro de pacientes morrendo por falta de oxigênio hospitalar, cuja carência e colapso foram previamente anunciados às autoridades estaduais e federais, estarreceu o país e o mundo.

O uso de máscaras e a prática do distanciamento social, que podem seguir progressivamente com o fechamento de escolas e universidades; a proibição de eventos de massa e de aglomerações; a restrição de viagens e transportes públicos; a conscientização da população para que permaneça em casa; e até a completa proibição da circulação nas ruas, exceto para a compra de alimentos e medicamentos ou a busca de assistência à saúde, ainda são essenciais^8^. Mas as vacinas são, até o momento, a estratégia mais promissora desde o início da epidemia. Alguns países desenvolvidos que conseguiram atingir altas taxas de coberturas vacinais com o uso de uma diversidade de estratégias, como Israel, obtiveram resultados iniciais animadores. Os dados sugerem que, mesmo diante de coberturas que não atingem 100% da população, os resultados podem ter marcantes reduções dos casos graves e de infecções. Em Israel, a taxa de infecção entre aqueles adequadamente vacinados, ou seja, uma semana após tomarem duas doses da vacina contra a doença, foi de 0,04%. Das pessoas vacinadas que foram infectadas, somente 16 tiveram que ser tratadas no hospital, ou seja, 0,002% do total^9^.

O objetivo deste estudo é analisar a situação da epidemia da covid-19 na região Nordeste do Brasil e discutir as possibilidades do seu comportamento diante do quadro político e econômico, da nova cepa, das medidas de distanciamento social e da disponibilidade das vacinas.

## MÉTODOS

O estudo foi realizado na região Nordeste do Brasil, uma das mais pobres do país. Os dados para os nove estados analisados são referentes ao período do início da epidemia, em março de 2020, até a segunda quinzena de março de 2021. Os casos de covid-19 confirmados e os óbitos são provenientes do Ministério da Saúde e das secretarias estaduais, disponíveis em repositório de dados abertos^10^.

A série de casos e de óbitos foi analisada para as capitais e para os estados, a partir da agregação dos dados dos municípios, com base na semana epidemiológica. A análise da série do acumulado semanal de casos e de óbitos confirmados da covid-19 foi feita com emprego do aplicativo JoinPoint Trend Analysis^11^. Para descrever tendências lineares por período, a mudança percentual anual estimada (Annual Percentage Change – APC) é calculada para cada tendência, ajustando uma linha de regressão ao logaritmo natural do número de casos e usando como unidade de tempo a semana epidemiológica como uma variável regressora. Um APC negativo significa uma diminuição do número de casos, enquanto um resultado positivo denota um aumento. Os resultados foram considerados como tendo uma mudança estatisticamente significativa na tendência se os coeficientes de regressão estimados para a diferença nas inclinações tivessem um valor de P inferior a 0,05^11^.

Para análise do percentual diário da população dos municípios e estados que permanece em casa, utilizou-se o índice de isolamento social, construído pela empresa “In Loco”, a partir de dados diários de geolocalização capturados em dispositivos móveis a partir de 1º de fevereiro de 2020^12^.

## RESULTADOS

Em 64 semanas de pandemia, o Nordeste contabilizou 2.922.126 casos de covid-19 e 69.009 óbitos confirmados (dados de 31/03/2021), representando 22,9% dos casos e 21,5% dos óbitos do país e apresentando, ao longo desse período, expressiva variabilidade no tempo e no espaço.

A tendência do número de casos confirmados e notificados dos nove estados da região Nordeste e suas respectivas capitais (Figuras 1 e 2), apesar de importantes variações, apresenta um pico inicial comum, que aconteceu por volta da 17ª semana da pandemia, e uma segunda fase de crescimento para um novo pico, que ainda não se completou, mas que na maioria dos estados e capitais já é ou será maior que o primeiro. No início da pandemia, período em que ocorreram os primeiros casos confirmados e óbitos pela doença, todos os estados apresentaram um crescimento sustentável de aproximadamente seis semanas, primeiro nas capitais, depois no interior. Em seguida, houve decréscimos em todos os estados e suas capitais, porém muitos permanecem com número de casos elevados, nunca atingindo um baixo patamar, antes da nova onda de crescimento. Em meados do segundo semestre de 2020, em todos os estados e suas capitais, o número de casos começou a crescer, em alguns locais com velocidade explosiva, em especial com a proximidade do final de 2020 e início de 2021, como ocorreu nos estados do Ceará, Pernambuco e Bahia. Quanto aos óbitos (Figuras 3 a 4), padrão similar ao de casos é observado, destacando-se que na maioria dos estados ou suas capitais o número já ultrapassou ou aproxima-se do teto observado na primeira onda.

**Figura 1:** Casos confirmados de covid-19 nas capitais dos nove estados da região Nordeste de acordo com o período e mudança estatisticamente significante do comportamento no tempo, por semana epidemiológica, 2020.

**Figura 2:** Casos confirmados de covid-19 nos nove estados da região Nordeste, de acordo com o período e mudança estatisticamente significante do comportamento no tempo, por semana epidemiológica, 2020.

**Figura 3:** Óbitos por covid-19 nas capitais dos nove estados da região Nordeste de acordo com o período e mudança estatisticamente significante do comportamento no tempo, por semana epidemiológica, 2020.

**Figura 4:** Óbitos por covid-19 nos nove estados da região Nordeste, de acordo com o período e mudança estatisticamente significante do comportamento no tempo, por semana epidemiológica, 2020.

Na primeira onda, todas as capitais e estados do Nordeste adotaram medidas de isolamento de forma mais intensa, com o *lockdown*, decretado quase simultaneamente nos nove estados. Fortaleza, Recife e Teresina atingem os maiores índice de isolamento de todas as capitais, perto de 0,60. Esse foi o único momento da epidemia em que capitais e estados conseguiram se aproximar de tais valores. Esse índice de isolamento começa, então, a decrescer, com algumas variações, atingindo seu mínimo (0,35) em torno do dia 9 outubro de 2020. A partir daí, o índice de isolamento tem uma tendência leve de crescimento, atingindo o pico em torno do dia 31 dezembro e passando a decrescer, com um pequeno pico de isolamento em meados de fevereiro, para depois decrescer novamente, com exceção de Fortaleza e Salvador, que permanecem com o índice de isolamento acima de 0,40. Todas as demais capitais, desde meados de fevereiro, caíram para menos de 0,40.

**Figura 5:** Medidas de distanciamento físico para estados e capitais do Nordeste.

## DISCUSSÃO

No início de 2021, vários estados do Nordeste passaram a apresentar aumento sustentado no número de casos e óbitos pela covid-19, e alguns, perigosamente, se aproximam de níveis muito próximos ou mesmo ultrapassam os valores da primeira onda. Nesta, houve ação mais organizada das autoridades estaduais e municipais da região, implementando medidas não farmacológicas e fortalecendo o sistema de saúde, que conseguiram amenizar os efeitos da epidemia numa região empobrecida^13^. Entretanto, na segunda onda, estados e municípios, embora uníssonos quanto aos esforços para ter a população vacinada o mais rapidamente possível, atuaram por meio de diferentes estratégias em outras áreas, com a maioria mantendo a economia totalmente aberta por um longo período, mesmo diante das evidências que se acumulavam de um agravamento do quadro epidêmico.

A abertura da economia no segundo semestre de 2020, sem o acompanhamento de medidas não farmacêuticas de enfrentamento do crescimento de casos, observados em muitas capitais e no interior já em finais de setembro, possivelmente explica o quadro atual, que rapidamente vem se tornando dramático, em especial para as populações mais vulneráveis^14^. Em novembro (semana epidemiológica 46) ocorreram as eleições, que já haviam sido adiadas por causa da covid-19^15^, nos 5.570 municípios brasileiros. Dessa forma, o período pré-eleições e a própria eleição, em muitos locais, promoveram aglomerações e maior possibilidade de disseminação do vírus^16^.

Além da abertura econômica e das eleições, juntaram-se as festas do final do ano e outro agravante que se impôs à pandemia no Brasil e no Nordeste: o surgimento de uma nova variante viral do Sars-Cov-2, denominada P1. A linhagem P1 foi primeiramente detectada em quatro viajantes que retornavam ao Japão procedentes do estado do Amazonas em 2 de janeiro de 2021^17^. Estima-se que tenha emergido em Manaus em meados de novembro de 2020, cerca de um mês antes do número de internações por síndrome respiratória aguda grave na cidade dar um salto. A nova cepa acumulou 17 mutações, até conseguir “escapar” da imunidade daqueles que já tiveram a doença^18,19^. Esta seria a provável explicação para o imenso surto ocorrido em Manaus no início deste ano. Deve-se ressaltar que Manaus já havia sofrido uma primeira onda bastante violenta e grande parte de sua população havia sido exposta à infecção pelo vírus^20^. A P1 consegue produzir reinfecções^6^, é cerca de 2,5^21^ a 10 vezes mais transmissível que as cepas originais^22^ e pode apresentar mortalidade maior que as cepas predecessoras, a exemplo da encontrada para a variante de preocupação identificada no Reino Unido, cuja mortalidade é 1,64 (IC95%: 1,34 – 2,04) vezes maior que a cepa anterior^23^.

A variante P1, como outras já descritas, aumenta a velocidade de transmissão (Rt), fazendo com que a epidemia tenha uma taxa maior de propagação e atinja mais pessoas em menos tempo. Também tem a capacidade de substituir a cepa anterior em um curto intervalo de tempo, cerca de sete semanas. Em Manaus, uma taxa de ataque estimada de 70% na primeira onda^24^ não impediu que ocorresse uma nova e devastadora onda, o que levou ao caos o frágil sistema de saúde local. Nenhuma ação, discutida com especialistas para conter a disseminação da nova variante do vírus detectada em Manaus, foi tomada. Ao contrário, porque o governo não promoveu a assistência necessária, doentes foram transferidos para diversos outros estados, sem cuidados especiais de segurança, o que pode ter contribuído para a disseminação da variante de Manaus para vários pontos do país.

A postura negacionista do governo federal deu uma grande contribuição para o caos que se instalou em Manaus e que está ocorrendo na maior parte do país, no Nordeste e em regiões mais desenvolvidas, como o Sul^25,26^. Uma pesquisa sobre as normas produzidas pelo atual governo federal relacionadas à pandemia de covid-19 revela uma estratégia institucional que, em vez de buscar controlar, favorece a propagação do vírus^27^. De fato, com o terreno pavimentado pela intensa redução das medidas de distanciamento e de uso de máscaras e o aumento da transmissão, a variante P1 rapidamente se disseminou por diferentes áreas do país. Estudo divulgado em início de março e realizado em oito estados brasileiros, três do Nordeste, identificou que 71,1% das amostras do Ceará, 50,8% das de Pernambuco e 42,6% das de Alagoas já eram da variante P1^28^.

À medida que se assiste à redução das medidas preventivas, como uso de máscaras, distanciamento social e etiquetas de higiene iniciadas em meados do segundo semestres de 2020, e novas variantes se espalham pelo estados, fica cada vez mais evidente a extensão da tragédia, com o colapso ou iminente colapso do sistema de saúde de várias cidades do país e da região Nordeste. Atingiu-se o ponto em que 25 das 27 capitais apresentam taxas de ocupação das UTI iguais ou superiores a 80%, com 15 delas já superando os 90%^29^, e o quadro só se agrava. A sobrecarga e o colapso do sistema de saúde são iminentes e o agravamento e “rejuvenescimento” da epidemia no Brasil são visíveis. A comparação do número de óbitos do período entre setembro e novembro de 2020 com o presente momento de colapso, entre 1º de fevereiro e 26 de março de 2021, realizado pela Associação de Medicina Intensiva Brasileira, mostra que as mortes na população entre 18 e 45 anos cresceu 193%, passando de 13,1% para 38,5% em UTI públicas e privadas do país^30^.

O quadro se agrava ainda mais por causa das baixíssimas taxas de cobertura vacinal. Embora o Brasil seja um dos únicos países capazes de vacinar 10 milhões de pessoas em um único dia, devido à alta qualidade e capilaridade do seu Programa Nacional de Imunização, vacinou apenas 8% da população^31^. O governo federal não somente deixou de comprar as vacinas necessárias, como também não organiza o processo de vacinação, e adicione-se que ainda desmerece o valor das vacinas disponíveis. O governo brasileiro poderia ter no primeiro semestre de 2021 em torno de 316 milhões de doses, das quais 200 milhões viriam da Covax^32^, Aliança Mundial de Vacinas formada por 165 países. Esse número seria suficiente para vacinar 78% da população, mas até o momento o país apenas começou a imunizar o segundo grupo dos quatro que integram as populações prioritárias, no qual estão inclusos os profissionais da saúde e idosos acima de 75 anos^33^. Pequenos avanços na vacinação mostraram queda da mortalidade e casos graves na população idosa vacinada em São Paulo (51%), Pernambuco (26%) e Ceará (46%)^34-36^. A busca por amenizar a situação, e uma ação articulada dos governadores do Consórcio Nordeste, levou à compra de 39 milhões de doses da vacina Sputnik V, desenvolvida na Rússia^37^. Esta vacina encontra-se em processo de aprovação para uso emergencial pela Agência Nacional de Vigilância Sanitária (Anvisa).

O cenário do Brasil não é bom e a situação do Nordeste é de alerta. As medidas dificilmente terão grande impacto se forem tratadas apenas regionalmente. Terão, sim, que ir para o âmbito nacional, e de forma muito mais efetivas, para enfrentar a pior crise sanitária, que chega no seu momento mais crítico. Configurando um dos piores indicadores da covid-19 do mundo, os números atuais podem ser atribuídos à falta de respostas efetivas e coordenas do governo federal à pandemia. Apesar de o Brasil apresentar um dos quadros mais graves e críticos da pandemia, somente um ano após do seu início o governo federal criou um Comitê de Coordenação Nacional para Enfrentamento da Pandemia da Covid-19^38^. Inúmeras dúvidas são levantadas sobre o real papel que este comitê pode representar no controle da epidemia no país, uma vez que ele é, em última instância, dirigido por um presidente negacionista.

A Frente Nacional de Prefeitos (FNP) afirmou em um documento que os brasileiros “estão abandonados pelo governo federal”^39^, ao mesmo tempo que o número de casos no Brasil cresce assustadoramente, e mais de 300 mil vidas se perderam, das quais 75% poderiam ter sido salvas^40^. Uma classificação criada para comparar o desempenho médio da gestão da pandemia de covid-19 ao longo do tempo em 98 países registrou que, até as 36 semanas após o centésimo caso confirmado do vírus, o Brasil ficou na 98ª posição, com o mais baixo score, de 4,8 em 100^41^. Outros agravantes, como o crescimento da insegurança alimentar por causa da pandemia, que, por exemplo, aumentou em 15,5% nas famílias com crianças de até 9 anos no estado do Ceará^42^, hão de tornar o quadro social e econômico cada vez mais complexos. São urgentes as implementações de medidas para proteger as populações mais vulneráveis, que engrossaram o percentual de pessoas em situação de pobreza.

Acelerar o processo de vacinação é medida imprescindível para reduzir a mortalidade, os casos graves da doença e mesmo os casos de covid-19 em geral. Dada a lentidão desse processo, conter a pandemia antes que a vacinação esteja em estágio avançado exigirá renovados esforços a fim de manter as medidas não farmacológicas existentes: constante uso de máscaras faciais, medidas de distanciamento e cuidados de higiene, além de políticas de proteção aos trabalhadores que perderam suas rendas e subsídios aos pequenos empresários.
